# Spectroscopic Behavior of Some A_3_B Type Tetrapyrrolic Complexes in Several Organic Solvents and Micellar Media

**DOI:** 10.3390/ijms12095552

**Published:** 2011-08-30

**Authors:** Rica Boscencu, Mihaela Ilie, Radu Socoteanu

**Affiliations:** 1 Faculty of Pharmacy, “Carol Davila” University of Medicine and Pharmacy, 6 Traian Vuia Street, Bucharest 020956, Romania; E-Mail: rboscencu@yahoo.com; 2 “Ilie Murgulescu” Institute of Physical Chemistry, Romanian Academy, 202 Splaiul Independenţei, Bucharest 060021, Romania; E-Mail: psradu@yahoo.com

**Keywords:** unsymmetrical mesoporphyrinic complexes, micelles, solvatochromy, spectroscopy

## Abstract

The paper presents spectral studies of some unsymmetrical A_3_B tetrapyrrolic, porphyrin-type complexes with Cu(II) and Zn(II) in different solvents and micellar media aimed at estimating their properties in connection with the living cell. The results indicate that the position of the absorption and emission peaks is mostly influenced by the central metal ion and less by the environmental polarity or the peripheric substituents of the porphyrinic core. The comparison between the overall absorption and emission spectra of the compounds in methanol or cyclohexane *vs.* direct and reverse Triton X micellar systems, respectively, suggests for all compounds the localization at the interface between the polyethylene oxide chains and the tert-octyl-phenyl etheric residue of the Triton X-100 molecules. These findings could be important when testing the compounds embedded in liposomes or other delivery systems to the targeted cell.

## 1. Introduction

Spectroscopic characterization of porphyrins in different media is an important step in assessing their application in the biomedical field, as the matrix in which they are supposed to act is a complex one, involving both hydrophilic and lipophilic characteristics [1–7].

The amphiphilic character of the porphyrins, given by the hydrophobic and hydrophilic groups placed in various proportions and positions of the substituted structure, generates an intramolecular axis of polarity. This specific structure is responsible for the biological properties of porphyrins related to the cell membranes penetration and/or cellular target binding. When used in therapy (e.g., photodynamic therapy—PDT) or as markers (molecular probes in photodynamic diagnosis—PDD), their spectral properties are important characteristics that often define their efficiency as photosensitizers [8,9]. Because the lifetime of oxygen singlet (^1^Δ_g_) in organic solvents or micelles is higher than in water, the singlet-oxygen-mediated photodamage will increase when the porphyrins are located in hydrophobic regions [10]. Also, the incorporation of porphyrins in micelles dramatically influences the aggregation characteristics and location of these molecules within the cells [11]. These are the main reasons to study the particular case of the A_3_B unsymmetrically *meso*-substituted metalloporphyrins as amphiphilic structures in different polarity media and incorporated in nanostructures such as micelles [12–14].

Solvatochromy studies can also provide valuable information to identify the potential molecular targets within the cell [10], to predict the cellular intake and subsequent porphyrin metabolism [15] and to obtain optimum conditions for the photosensitization, as spectral properties are often dramatically changed by the microenvironment [16,17].

**Figure 1:**
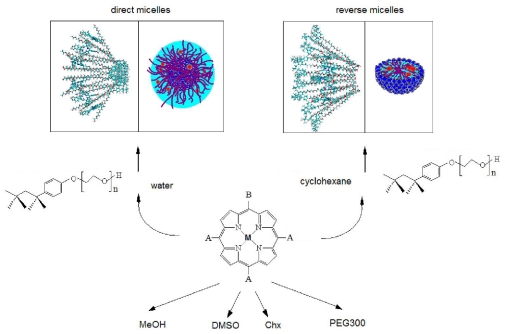
General scheme of the porphyrinoid systems studied.

The non-ionic surfactants exhibit an extended polar part, with variable dimensions given by the number of oxyethylene units, which is highly influenced by hydration. Among this kind of surfactants, *tert*-octylphenoxypolyethoxyethanol (Triton X-100) has a polyoxyethylene chain consisting of 9.5 ethylene oxide units on average, thus being a medium-chain length surfactant [18]. Literature data indicate a general good understanding of the rules governing self-assembly in direct (DM) and reverse micelles (RM) built with this surfactant [19,20]. Therefore, we chose to work with two well characterized TX-100 micelle systems, having well-defined dimensions, aggregation numbers and core polarities (Figure 1). Polyethylene glycol (PEG) has been widely used in pharmaceutical preparations [21]. The similarity of PEG chemical structure allowed us to compare the spectral changes observed for the porphyrins embedded in DM and RM and to suggest the possible localization of the compounds tested within the micelles.

**Figure 2:**
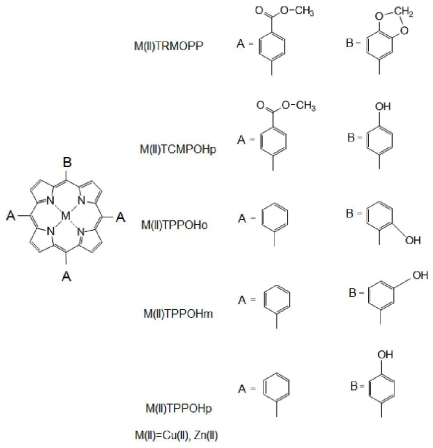
General structures of the unsymmetrical mesoporphyrinic complexes used in this study.

The study presents a few of such spectral properties for some unsymmetrical porphyrinic complexes which have been previously synthesized [22–24], and are presented in Figure 2: 5-[(3,4- methylendioxy)phenyl]-10,15,20-*tris*-(4-carboxymethylphenyl)-21,23-Zn(II)-porphine (Zn(II)TRMOPP), 5-[(3,4-methylendioxy)phenyl]-10,15,20-*tris*-(4-carboxymethylphenyl)-21,23-Cu(II)-porphine (Cu(II) TRMOPP), 5-(4-hydroxyphenyl)-10,15,20-*tris*-(4-carboxymethylphenyl)-21,23-Zn(II)-porphine (Zn(II)TCMPOHp), 5-(4-hydroxyphenyl)-10,15,20-*tris*-(4-carboxymethylphenyl)-21,23-Cu(II)-porphine (Cu(II)TCMPOHp), 5-(x-hydroxyphenyl)-10,15,20-*tris*-phenyl-21,23-Cu(II)-porphine (Cu(II)TPPOH_X_), 5-(x-hydroxyphenyl)-10,15,20-*tris*-phenyl-21,23-Zn(II)-porphine (Zn(II)TPPOH_X_) (x = 2, 3, 4).

The spectral characteristics defined as follows could predict the spectral characteristics of protein-bound mesoporphyrinic systems and also the properties of the studied compounds in pharmaceutical formulation.

## 2. Results and Discussion

### 2.1. UV-Vis Spectroscopy

Porphyrinic complexes display in their molecular absorption spectra, one Soret (B) band as a result of a_1u_ (π) → e_g_ (π*) transition, generally situated in the spectral range 400–440 nm, and one or two Q bands between 500–650 nm, corresponding to the a_2u_ (π) → e_g_ (π*) transition [25,26].

**Figure 3:**
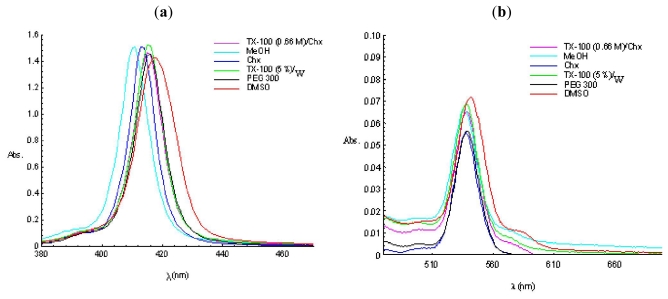
Absorption spectra of 2.5 × 10**^−^**^6^ M Cu(II)TPPOH_m_ in different solvents and micellar solutions: (**a**) Soret band; and (**b**) Q band.

The unsymmetrical mesoporphyrinic complexes used in this study revealed a strong Soret band with maximum in the range 410–430 nm accompanied by Q bands situated in the range 540–600 nm (Figure 3, Tables 1, 2). Results are consistent with those obtained on similar compounds [27].

The main differences observed in the spectral band position are due to the type of metallic ion. That means that the influence of the central ion is higher in what concerns the displacement of the spectral bands position compared to that induced by the peripheral substituents of the porphyrinic macrocycle, which is of only 2–4 nm. Therefore, in the case of Cu(II) complexes, Soret bands are 10–12 nm hypsochromically shifted compared to the corresponding Zn(II) complex having the same ligand, in the same solvent. Similarly, the Q bands of the copper porphyrins show a blue shift of about 20 nm compared to the corresponding zinc porphyrins. Results confirm earlier results obtained with other metalloporphyrins [22–24,26].

These spectral differences are the result of stronger conjugation effects that occur between the metallic ion orbitals and the π electrons of the tetrapyrrolic ring, effects that cause an energy decrease of the a_1u_ (π) and a_2u_ (π) orbitals relative to the e_g_ (π*) orbitals, with increased energy available for copper porphyrins that generate the blue shift of the spectral bands compared to the zinc porphyrins [28].

From the experimental data we could observe that the influence of the non-symmetrical substituents *versus* spectral properties of the metalloporphyrins is insignificant. The hydrophilic groups placed in various positions of the porphyrinic structure did not significantly disturb the inner π electron ring of the macrocycle, which is responsible for the active electronic transitions in the above mentioned spectral range [29,30].

The change of the charge density and distribution on the periphery of the porphyrine macrocycle induced by the polar substituents supports the localization of the photosensitizer at the cellular level, without significant change of the spectral properties responsible to its photophysical activation.

The organic solvents were chosen for the study taking into account their relative polarity, as defined by Reichard [31]. Thus, cyclohexane, dimethysulfoxide and methanol, having relative polarity indices of 0.0006, 0.444 and 0.762, respectively, have been chosen. Only for comparison purposes, the ethylene glycol (relative polarity index 0.790) was also chosen.

The spectral data obtained indicate that the position of the absorption bands is less influenced by environmental polarity. However, a few observations can be made. For the same compound, the longest wavelengths maxima can be found in dimethylsulfoxide (generally, about 5 nm bathochromically shifted as compared to the other solvents), followed by cyclohexane and methanol (with 1 to 3 nm separate from each other). The observation is valid for both Soret and Q bands.

In case of direct micelles compared to PEG 300 (considered as reference), the shifts are too small (1–2 nm) or missing (in most cases in copper porphyrinic complexes). Meanwhile, the comparison of the wavelengths of the maxima with those belonging to the same compound dissolved in methanol (the most polar solvent) show that there is a slight bathochromic shift in case of the direct micelles *vs.* MeOH, suggesting the presumptive localization for the entire group of porphyrins as individual probe in direct micelles, as expected, in the polyethyleneoxide chain, at the interface with the *tert*-octyl-phenylether bulk of the TX-100 molecule, and not at the water-oxyethylene chain interface.

Similar results were obtained in the case of reverse micelles, *i.e.*, the maxima of the Soret bands position are in the same region as the PEG 300 reference system, indicating the metalloporphyrins localization at the oxyethylene chains level. The results confirm those obtained earlier on similar mesoporphyrinic compounds [32].

### 2.2. Fluorescence Spectroscopy

The fluorescence measurements were performed on the studied complexes in the same solvents and micellar systems. Only the zinc porphyrinic compounds exhibit fluorescent properties strong enough to be taken into account.

In order to get a good comparison between the compounds, the excitation was set to 420 nm.

**Figure 4:**
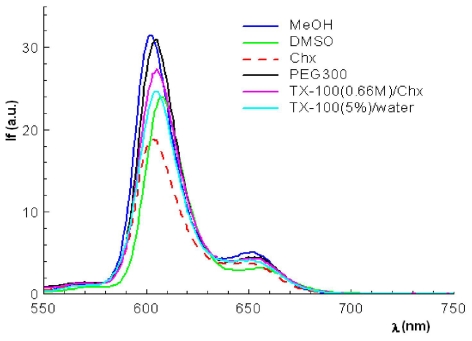
Fluorescence emission of 5-(4-hydroxyphenyl)-10,15,20-*tris*-phenyl-21, 23-Zn(II)-porphine in different solvents and micellar solutions (*c* = 2.5 × 10^−6^ M, λ_ex_ = 420 nm).

One can observe that the differences between the emission wavelengths are small, confirming the results already obtained for the absorption spectra. Therefore, the fluorescence spectral data shows for zinc porphyrinic complexes two bands located in the spectral region of 605–665 nm (Figure 4, Table 3) and reveal smaller shifts of the emission maxims when changing the environmental polarity. This smal bathocromic shift of the fluorescence maximum position indicates incorporation of the porphyrinic complex in the micellar media.

In Triton X-100/cyclohexane reverse micelles the fluorescence data of zinc complexes confirm their localization in the area of the polyethyleneoxidic chains, as the maximum wavelength is located in the same spectral range as in the PEG 300 reference system.

For the zinc tetrapyrollic complexes a smaller bathochromic shift in direct micelles was registered as compared to PEG 300 systems and methanolic solution, suggesting a localization of the complex in the polar area of the micelle.

The results indicate that the administration of the porphyrins to cells in view of photosensitization will probably not change the photophysical characteristics of the porphyrins even embedded in liposomes or other kind of delivery systems.

The Stokes shifts of the studied Zn(II) mesoporphyrins, computed as the difference between the maximum of emission and that one of absorption, as displayed in Table 4, show that the influence DMSO compared to the other two solvents used results in less change of the Stokes shift (of 1 to 3 nm).

## 3. Experimental Section

Porphyrinic complexes used in this study were synthesized as previously described [22–24].

Poly (oxyethylene) tert-octylphenyl ether (Triton X-100–TX, purity > 99%), methanol (MeOH, HPLC gradient grade), dimethyl sulfoxide (DMSO, analytical grade) dichloromethane (analytical grade) and polyethylene glycol 300 (Carbowax 300) were bought from Sigma and used without supplementary preparation before. Water was double distilled and deionized before use.

Molecular absorption spectra were recorded on a Lambda 35 Perkin-Elmer UV-Vis spectrophotometer in 10 mm path length quartz cells, in single beam mode.

Fluorescence spectra were recorded on a steady-state Jasco FP 6500 spectrofluorimeter in 10 mm path length quartz cells.

The solutions used were prepared by repeated dilution to obtain a final 2.5 × 10^−6^ M concentration of each compound in the three solvents.

The 0.24 mM TX-100 in water (w/w) direct micelles (DM) and 0.66 M TX-100 in cyclohexane (w/w) reverse micelles (RM) loaded with 2.5 × 10^−6^ M metalloporphyrins were prepared according to the procedure described before [33]. Briefly, appropriate volumes of metalloporphyrins in dichloromethane solutions were evaporated to dryness at room temperature on the bottom of a test tube. Aliquots of 3mL of appropriate concentrations of Triton X-100 in water and cyclohexane were added, and then the tubes were mildly vortex mixed for 5 minutes, capped and then left still overnight to ensure the solubilization and diffusion of the metalloporphyrins into the micelles. The final concentration of each porphyrinic compound in micellar media was set at 2.5 × 10^−6^ M; the solutions were kept in dark to prevent photodegradation before the measurements, which were performed 24 h after preparation.

## 4. Conclusions

The paper presents the molecular spectral characteristics of certain A_3_B type *meso*-substituted metalloporphyrins in different polarity solvents and in micellar media. The studies revealed that the position of Soret and Q bands of the porphyrins is mainly influenced by the central metal ion and less by the peripheral substituents of the porphyrinic core or polarity of the solvent, thus confirming the results previously obtained on other metalloporphyrins.

The estimated localization of the porphyrinic in direct and reversed micelles compounds was possible by the evaluation of the spectral changes, for each compound, using PEG 300 as reference *versus* TX-100 (0, 66 M)/cyclohexane and TX-100 (5% g/g)/water, accounting simultaneously for the micropolarity of the oxyethylenic chains and of the hydrophilic and hydrophobic parts of micellar systems. For all compounds, the localization at the interface between the polyethylene oxide chains and the *tert*-octyl-phenyl etheric residue of the Triton X-100 molecules was considered most probable. The steady-state fluorescence emission studies confirm the results obtained by UV-Vis absorption in what concerns the small differences between the maximum emission peak in correlation to the chemical structure of the porphyrin and in the lack of correlation between the position of the bands and the solvent polarity as defined by Reichard. All these findings suggest that the photophysical properties of the compounds will not be dramatically changed if the compounds are embedded in liposomes to obtain a better delivery to the cellular target.

## Figures and Tables

**Table 1:** Wavelengths maxima (λ_max_) and molar extinction coefficient values (lg ɛ) for the zinc porphyrinic complexes in different solvents and micellar media (*c* = 2.5 × 10^−6^ M).

Solvent	λmax (nm) [lg ɛ (L mol^−1^ cm^−1^)]		
	Soret B (0,0)	Q (1,0)	Q (0,0)

***5-(2-hydroxyphenyl)-10,15,20-tris-phenyl-21,23-Zn(II)-porphine***			
**MeOH**	421 [5.726]	556 [4.342]	595 [4.033]
**DMSO**	428 [5.695]	560 [4.326]	560 [4.121]
**Chx**	425 [5.570]	557 [4.191]	597 [3.741]
**PEG300**	426 [5.659]	558 [4.310]	597 [4.000]
**TX/water**	427 [5.674]	559 [4.357]	598 [4.342]
**TX/Chx**	427 [5.674]	559 [4.356]	598 [4.334]

***5-(3-hydroxyphenyl)-10,15,20-tris-phenyl-21,23-Zn(II)-porphine***			
**MeOH**	421 [5.867]	556 [4.394]	595 [3.982]
**DMSO**	428 [5.867]	559 [4.408]	600 [4.146]
**Chx**	425 [5.778]	557 [4.254]	596 [3.702]
**PEG300**	426 [5.820]	558 [4.358]	598 [4.000]
**TX/water**	426 [5.817]	559 [4.471]	599 [4.342]
**TX/Chx**	427 [5.991]	558 [4.408]	598 [4.017]

***5-(4-hydroxyphenyl)-10,15,20-tris-phenyl-21,23-Zn(II)-porphine***			
**MeOH**	422 [5.718]	557 [4.246]	596 [3.924]
**DMSO**	429 [5.702]	561 [4.246]	602 [4.017]
**Chx**	425 [5.729]	558 [4.292]	598 [3.903]
**PEG300**	427 [5.713]	559 [4.350]	599 [4.121]
**TX/water**	428 [5.709]	560 [4.255]	601 [3.982]
**TX/Chx**	427 [5.713]	560 [4.265]	600 [3.964]

***5-[(3,4-methylendioxy)phenyl]-10,15,20-tris-(4-carboxymethylphenyl)-21,23-Zn(II)-porphine***			
**MeOH**	424 [5.686]	557 [4.255]	598 [3.880]
**DMSO**	431 [5.632]	562 [4.246]	603 [3.964]
**Chx**	425 [5.554]	558 [4.218]	599 [3.941]
**PEG300**	427 [5.577]	559 [4.221]	599 [3.940]
**TX/water**	428 [5.584]	559 [4.224]	602 [3.962]
**TX/Chx**	427 [5.579]	559 [4.226]	602 [3.963]

***5-(4-hydroxyphenyl)-10,15,20-tris-(4-carboxymethylphenyl)-21,23-Zn(II)-porphine***			
**MeOH1**	425 [5.732]	558 [4.342]	599 [4.049]
**DMSO1**	431 [5.800]	563 [4.246]	604 [4.028]
**Chx**	427 [5.571]	561 [4.255]	601 [3.826]
**PEG300**	426 [5.640]	558 [4.272]	599 [3.956]
**TX/water**	427 [5.570]	559 [4.232]	600 [3.980]
**TX/Chx**	426 [5.616]	558 [4.265]	598 [3.856]

MeOH = methanol; DMSO = dimethylsulfoxide; Chx = cyclohexane; PEG300 = polyethylenegliycol 300; TX = Triton X-100;

1 Data were taken from [22].

**Table 2:** Wavelengths maxima (λ_max_) and molar extinction coefficient values (lg ɛ) for the copper porphyrinic complexes in different solvents and micellar media (*c* = 2.5 × 10^−6^ M).

Solvent	λmax (nm) [lg ɛ (L mol^−1^ cm^−1^)]	
	Soret B (0,0)	Q (1,0)

***5-(2-hydroxyphenyl)-10,15,20-tris-phenyl-21,23-Cu(II)-porphine***		
**MeOH**	411 [5.820]	537 [4.447]
**DMSO**	417 [5.769]	540 [4.505]
**Chx**	413 [5.843]	538 [4.447]
**PEG300**	416 [5.763]	539 [4.380]
**TX/water**	415 [5.838]	538 [4.505]
**TX/Chx**	416 [5.867]	539 [4.447]

***5-(3-hydroxyphenyl)-10,15,20-tris-phenyl-21,23-Cu(II)-porphine***		
**MeOH**	411 [5.780]	536 [4.422]
**DMSO**	418 [5.757]	542 [4.422]
**Chx**	414 [5.782]	538 [4.350]
**PEG300**	416 [5.763]	538 [4.350]
**TX/water**	416 [5.784]	539 [4.441]
**TX/Chx**	415 [5.766]	538 [4.415]

***5-(4-hydroxyphenyl)-10,15,20- tris-phenyl - 21,23-Cu(II)-porphine***		
**MeOH**	412 [5.701]	538 [4.422]
**DMSO**	420 [5.695]	542 [4.428]
**Chx**	414 [5.678]	539 [4.394]
**PEG300**	417 [5.684]	540 [4.326]
**TX/water**	416 [5.691]	539 [4.441]
**TX/Chx**	417 [5.701]	539 [4.537]

***5-[(3,4-methylendioxy)phenyl]-10,15,20-tris-(4-carboxymethylphenyl)-21,23-Cu(II)-porphine***		
**MeOH**	414 [5.741]	538 [4.394]
**DMSO**	422 [5.652]	544 [4.408]
**Chx**	416 [5.770]	540 [4.371]
**PEG300**	417 [5.782]	540 [4.803]
**TX/water**	418 [5.792]	539 [4.800]
**TX/Chx**	418 [5.788]	540 [4.807]

***5-(4-hydroxyphenyl)-10,15,20-tris-(4-carboxymethylphenyl)-21,23-Cu(II)-porphine***		
**MeOH1**	414 [5.532]	539 [4.260]
**DMSO1**	423 [5.507]	545 [4.203]
**Chx**	419 [5.661]	540 [4.283]
**PEG300**	416 [5.782]	539 [4.150]
**TX/water**	416 [5.507]	539 [4.440]
**TX/Chx**	416 [5.766]	539 [4.415]

MeOH = methanol; DMSO = dimethylsulfoxide; Chx = cyclohexane; PEG300 = polyethylenegliycol 300; TX = Triton X-100;

1 Data were taken from [22].

**Table 3:** Fluorescence emission peak wavelength of the zinc mesoporphyrinic complexes in solvents with different polarities and micellar media (*c* = 2.5 × 10^−6^ M, λ_ex_ = 420 nm).

Solvent	λmax (nm)	
***5-(2-hydroxyphenyl)-10,15,20-tris-phenyl-21,23-Zn(II)-porphine***		
**MeOH**	600	651
**DMSO**	605	654
**Chx**	602	651
**PEG300**	602	651
**TX/water**	603	652
**TX/Chx**	602	651
***5-(3-hydroxyphenyl)-10,15,20-tris-phenyl-21,23-Zn(II)-porphine***		
**MeOH**	600	650
**DMSO**	605	656
**Chx**	605	656
**PEG300**	602	652
**TX/water**	603	652
**TX/Chx**	603	651
***5-(4-hydroxyphenyl)-10,15,20-tris-phenyl-21,23-Zn(II)-porphine***		
**MeOH**	602	651
**DMSO**	608	655
**Chx**	602	652
**PEG300**	605	653
**TX/water**	605	653
**TX/Chx**	605	651
***5-[(3,4-methylendioxy)phenyl]-10,15,20-tris-(4-carboxymethylphenyl)-21,23-Zn(II)-porphine***		
**MeOH**	605	652
**DMSO**	610	658
**Chx**	604	650
**PEG300**	605	662
**TX/water**	606	665
**TX/Chx**	605	665
***5-(4-hydroxyphenyl)-10,15,20-tris-(4-carboxymethylphenyl)-21,23-Zn(II)-porphine***		
**MeOH1**	605	652
**DMSO1**	611	656
**Chx**	604	652
**PEG300**	605	663
**TX/water**	606	665
**TX/Chx**	606	665

MeOH = methanol, DMSO = dimethylsulfoxide, Chx = cyclohexane, PEG300 = polyethylene glycol 300, TX = Triton X-100;

1 Data were taken from [22].

**Table 4:** Stokes shifts (λ_em_ – λ_abs_) for the Zn(II) complexes [nm].

Porphyrinic complexes	λ_em_ – λ_abs_ [nm]					
	MeOH	DMSO	Chx	PEG300	TX-100/w	TX-100/Chx
**Zn(II)TPPOHo**	179	177	177	177	176	175
**Zn(II)TPPOHm**	179	177	176	176	177	176
**Zn(II)TPPOHp**	180	179	177	178	177	178
**Zn(II)TRMOPP**	181	179	179	178	178	178
**Zn(II)TCMPOHp**	180	180	177	179	179	180
